# Patient's needs and preferences in routine follow-up after treatment for breast cancer

**DOI:** 10.1038/sj.bjc.6601655

**Published:** 2004-02-24

**Authors:** G H de Bock, J Bonnema, R E Zwaan, C J H van de Velde, J Kievit, A M Stiggelbout

**Affiliations:** 1 Department of Medical Decision Making, Leiden University Medical Center, Leiden, The Netherlands; 2 Department of Surgery, Leiden University Medical Center, Leiden, The Netherlands

**Keywords:** follow-up, patient care, informational needs, patient preferences

## Abstract

The purpose of the study was to analyse the needs of women who participated in a routine follow-up programme after treatment for primary breast cancer. A cross-sectional survey was conducted using a postal questionnaire among women without any sign of relapse during the routine follow-up period. The questionnaire was sent 2–4 years after primary surgical treatment. Most important to patients was information on long-term effects of treatment and prognosis, discussion of prevention of breast cancer and hereditary factors and changes in the untreated breast. Patients preferred additional investigations (such as X-ray and blood tests) to be part of routine follow-up visits. Less satisfaction with interpersonal aspects and higher scores on the Hospital Anxiety and Depression Scale (HADS) scale were related to stronger preferences for additional investigation. Receiving adjuvant hormonal or radiotherapy was related to a preference for a more intensive follow-up schedule. There were no significant differences between patients treated with mastectomy compared to treated with breast-conserving therapy. During routine follow-up after a diagnosis of breast cancer, not all patients needed all types of information. When introducing alternative follow-up schedules, individual patients’ information needs and preferences should be identified early and incorporated into the follow-up routine care, to target resources and maximise the likelihood that positive patient outcomes will result.

Routine follow-up programmes in specialist clinics are part of the standard medical care after treatment for primary breast cancer. Regularly scheduled follow-up visits are intended to detect and treat recurrence at an early stage and provide reassurance and psychological support to the patient (Tomiak and Piccart, 1993). There is much debate about how to organise cost-effective routine follow-up (Dewar, 1995). In current follow-up schedules in Western countries, patients are seen for follow-up about 12–17 times in the first 5 years after surgery for breast cancer (Schapira and Urban, 1991). These follow-up visits are time consuming and expensive for both the patient and the physician. Moreover, the effect of follow-up on overall survival rates remains highly questionable (GIVIO Investigators, 1994; Palli *et al*, 1999; te Boekhorst *et al*, 2001; Jacobs *et al*, 2001).

Given these considerations, alternative strategies in follow-up have been proposed, including the introduction of specialised breast-care nurses to perform the follow-up (Bryan *et al*, 2002), primary care involvement (Grunfeld *et al*, 1996), various forms of patient-initiated follow-up (Brown *et al*, 2002), and less-intensive and shorter follow-up schedules (Gulliford *et al*, 1997). A patient's perspective is an important factor in evaluating these various follow-up strategies.

In this paper, we focus on a patient's needs and preferences in routine follow-up 2–4 years after treatment for primary breast cancer. We studied the information needs and preferences regarding additional investigations and organisation of follow-up care. Furthermore, we analysed whether women with lower or higher information needs could be characterised in terms of attitudes towards and expected benefits from follow-up, psychological functioning, age, treatment for breast cancer, or duration of follow-up.

## METHODS

### Participants and follow-up schedule

This cross-sectional survey was performed in December 2001 among all 116 patients who were treated for early breast cancer between January 1998 and December 1999 in the Leiden University Medical Center in Leiden, the Netherlands. Patients had been treated by mastectomy or breast-conserving therapy (lumpectomy and postoperative irradiation) and had no signs of local, regional or distant relapse during the follow-up period. Adjuvant systemic treatment had been given according to national guidelines. Family risk assessment was a regular part of primary counselling and if applicable, patients were referred to the Department of Clinical Genetics. Such a referral did not alter the follow-up scheme regarding breast cancer.

During primary treatment (including adjuvant treatment), patients were informed verbally by their hospital doctor about the benefits and complications of therapies. Information on the goals of follow-up and on the investigations performed was not given in a structured way and there was no written information on these topics. In that time period, in our hospital there was no counselling or support of a specialist breast-care nurse available.

All patients participated in a standard routine follow-up schedule according to Dutch Association of Comprehensive Cancer Centers guidelines for follow-up (Vademecum Oncologie, 1992). This included visits every 3 months during the first 2 years, visits every 6 months during the next 3 years, and annual visits thereafter, for a maximum of 10 years. Follow-up visits were performed by a hospital doctor in the surgical oncology outpatient clinic. Patients treated by radiation or chemotherapy were also seen in the radiation or medical oncology clinic. As a consequence, patients were reviewed by several physicians during their follow-up. During each visit, a history was taken concerning symptoms that could signal relapse or metastases, and a standard physical examination was performed. Mammography was carried out at yearly intervals.

A final questionnaire was mailed to the home of all patients (*n*=116) accompanied with a letter that explained the purpose of the study. The questionnaire could be sent back in a self-addressed envelope. Initially, 63 patients returned the questionnaire. A total of 53 patients were sent a reminder, resulting in an additional 21 questionnaires returned. In total, 84 questionnaires were returned (response rate 72%). The nonresponders did not differ regarding age or received treatment (*P*=0.49).

### Measurements

Apart from general data concerning sociodemographic status and medical history, the postal questionnaire consisted of six sections that are outlined below. The ‘*Expected benefits from routine follow-up*’ and ‘*Needs and preferences in routine follow-up*’ were measured with a self-developed questionnaire, because existing questionnaires (Degner *et al*, 1997; Graydon *et al*, 1997; Mesters *et al*, 2001) were unsuitable for measuring specific needs and benefits in breast cancer routine follow-up. In a pilot study, the feasibility of the questionnaire was tested, for acceptability and understanding, among 15 women who were in follow-up at our breast clinic and who were treated for invasive breast cancer before January 1998.

#### Attitudes towards follow-up

Patients were asked to fill out a validated 15-item questionnaire on attitudes towards follow-up that had been used to evaluate the follow-up of colorectal cancer (Stiggelbout *et al*, 1997). This questionnaire consists of four subscales: communication (with the physician), reassurance, nervous anticipation, and specific perceived disadvantages of follow-up. For the communication and the reassurance scales, a higher score meant a more positive evaluation (range: 0–100). For the nervous anticipation and the disadvantages scales, a higher score meant more negative effects (range: 0–100).

#### Expected benefits from follow-up

Questions were included about the expected benefits, from a patient's perspective, regarding the purposes of breast self-examination, physical examination of the breasts by a doctor, mammography, and the patient's ideas about the curability of the disease after the detection of distant metastases (eight items in total). Patients could choose from four answering categories: not at all, somewhat, rather, very much. Scores were summed up and normalised to a 100-point scale (transformed score=((sum score–potential minimum of sum scores)/potential range of sum scores) × 100). A higher score meant more expected benefits (range: 0–100). The reliability (internal consistency) of the expected benefits from follow-up scale was high (0.79).

#### Satisfaction with oncological care

Patient satisfaction with oncological care was assessed using the Dutch version of Ware's Patient Satisfaction Questionnaire III (PSQ III) (Hagedoorn *et al*, 2003). This questionnaire (36 items) was designed to measure technical competence, interpersonal manner, and access to care. To get an impression of the general satisfaction, we also calculated an overall score on the PSQ III. Higher score means more satisfaction with the oncological care received (range: 0–100).

#### Quality of life and psychological functioning

A three-item scale assessed fear of recurrence. This scale has been used in several former studies at our hospital (Stiggelbout *et al*, 1997). The higher the score, the more the fear of recurrence (range: 0–100). The patients rated their overall quality of life during the preceding week in two ways, by means of a seven-point verbal scale (ranging from excellent to very bad (de Haes *et al*, 1990a); and by means of a visual analogue scale (a 100 mm horizontal line, anchored at the extremes by ‘best imaginable quality of life’ and ‘worst imaginable quality of life’ (de Haes *et al*, 1990b). Additionally, patients were asked to fill out the Dutch version of the HADS (Spinhoven *et al*, 1997). The higher the score, the more anxious and depressed the patient (range: 0–14).

#### Sociodemographic data and medical history

Registered was age at the time of filling in the questionnaire, stage of breast cancer (DCIS, I, IIA, IIB, or IIIA), treatment for breast cancer (mastectomy *vs* breast-conserving therapy; adjuvant hormonal therapy (yes or no), adjuvant chemotherapy (yes or no), adjuvant radiotherapy (yes or no)), and duration of follow-up (the period between operation and last follow-up visit).

#### Needs and preferences in routine follow-up

Needs of patients were subdivided into two main categories, the first addressing information needs, the second medical technical preferences with respect to follow-up. The topics are outlined in Table 2. They could chose between three answering categories: not important/do not wish this, not very important/do not care, very important/I certainly want this. In a factor analysis, two (orthogonal) factors or subscales were found: a factor containing needs for general topics and a factor containing needs for more specific topics. The reliability (internal consistency) of the first scale, containing needs on general topics, was 0.81 and of the second scale, containing needs on more specific topics, was 0.91. The explained variance was 27 and 15%, respectively. Per scale the scores were added up. The higher the score, the more needs there were.

With respect to medical technical aspects of follow-up, patients were asked how much they would like additional investigations (e.g. X-ray and blood tests) to be part of the follow-up-visit, how often they preferred to attend routine control visits (every 3 months, every 6 months, or every year), for how long they preferred to attend routine control visits (5 years, 10 years, or lifelong) and by whom the follow-up should be performed (general practitioner, specialised breast-care nurse, or hospital doctor). These four questions regarding preferences for additional investigations and organisation of routine follow-up care were considered separately.

### Analyses

The following patient characteristics were considered as possible determinants of needs and preferences: age, treatment for breast cancer, duration of follow-up, attitudes and expected benefits from follow-up, satisfaction with oncological care, quality of life and psychosocial functioning. Cutoffs for the Hospital Anxiety and Depression Scale (HADS) were based on Carroll *et al* (1993) For all scales, missing data were replaced by the individual mean for that scale, if no more than 50% of the items on the scale were missing. Otherwise, the scale value was considered as missing.

The answers relating to needs and preferences were described. Spearman's rank correlations, between needs and preferences on the one hand and the patient characteristics on the other, were calculated. For some (dicho- and trichotomous) variables, other methods (such as *χ*^2^) would have been more appropriate. However, these other methods rendered almost identical results and are therefore not reported. Finally, for the patient characteristics that correlated significantly with their needs or preferences, a multivariate model was constructed for each dependent variable.

## RESULTS

### Participants

For an overview of the patient characteristics, see Table 1

**Table 1:** Patient characteristics (*n*=84)

Age: median (range)	56 (33–90)	
*Stage of breast cancer*		
DCIS	11 (13%)	
I	36 (43%)	
IIA	17 (20%)	
IIB	17 (20%)	
IIIA	3 (4%)	
		
*Surgical therapy; n* (%)		
Breast-conserving therapy	32 (39 %)	
With adjuvant hormonal therapy	4 (12%)	
With adjuvant chemotherapy	6 (19%)	
With adjuvant radiotherapy	31 (97%)	
Mastectomy	51 (61 %)	
With adjuvant hormonal therapy	14 (27 %)	
With adjuvant chemotherapy	23 (45 %)	
With adjuvant radiotherapy	15 (29 %)	
Time since primary surgical therapy in years: median (range)	3.0 (2.0–4.1)	
		
Duration of follow-up in years: median (range)	3.0 (2.0–4.1)	
		
*Attitudes towards follow-up; median (range)*		
Communication (with the physician)	75 (0–100)	
Reassurance	50 (0–67)	
Nervous anticipation	17 (0–92)	
Specific perceived disadvantages of follow-up	11 (0–67)	
		
*Expected benefits from follow-up*	*Not at all/somewhat*	*Rather/very much*
To what extent you think that …	31%	69%
Physical examination will detect a new tumour in the other breast?	13%	87%
Mammography will detect a new tumour in the other breast?	50%	50%
Self-examination will detect a new tumour in your breast?		
Early detection of a new tumour in the other breast will contribute to your cure?	9 %	91%
Early detection of distant recurrences will contribute to your cure?	12%	88%
Physical examination will detect a new tumour in the operated breast?	17%	83%
Mammography will detect a new tumour in the operated breast?	10%	90%
Early detection of a tumour in the operated breast will contribute to your cure?	10%	90%
		
*Ware's Patient Satisfaction Questionnaire III: mean; s.e. (range)*		
Technical competence	73; 1.8 (35–100)	
Interpersonal aspects	76; 1.9 (39–100)	
Access to care	70; 1.8 (19–100)	
Total score	74; 1.6 (42–100)	
		
*Quality of life and psychological functioning*		
Fear of recurrence; median (range)	33 (0–100)	
Quality of life on a visual analogue scale; median (range)	80 (0–100)	
Quality of life on a verbal scale; median (range)	5 (1–7)	
HADS-anxiety; mean; s.e. (range)	5.0; 0.3 (0–13)	
HADS-depression; mean; s.e. (range)	2.7; 0.3 (0–14)	

. The median age of patients was 56 years (range 33–90). The median duration of follow-up was 3.0 years (range 2.0–4.1).

#### Attitudes towards follow-up

Regarding the attitudes towards follow-up, patients considered communication with the physician as rather positive (median score: 75) and obtained a moderate sense of reassurance from follow-up (median score: 50). They did not perceive the disadvantages to be large, nor did they indicate feeling much nervous anticipation (median scores: 11 and 17, respectively).

#### Expected benefits from follow-up

Patients had high-expected benefits from follow-up, especially from mammography and early detection of a breast cancer recurrence. Most patients (88%) believed that early detection of distant metastases would contribute to cure. Breast self-examination as an instrument of early detection of breast cancer was not highly valued.

#### Satisfaction with oncological care

Patients were rather satisfied with the care they received (median score: 74). They were most satisfied with interpersonal aspects (interpersonal manner and time spent with physician; median score: 76). Satisfaction regarding access to care was somewhat lower (median score: 70).

#### Quality of life and psychological functioning

Patients had a moderate fear of recurrence (median score: 33). They scored their quality of life well on a visual analogue scale and on a verbal scale (median score: 80 and 5,respectively). The mean scores on the HADS were 5.0 for anxiety, and 2.7 for depression. Using the previous mentioned cutoff score of 8 for the anxiety and depression subscales, we found that 18% of this population would warrant further psychiatric evaluation. A total of 7% had scores 11 or more and would be those most likely to have had anxiety (6%) or depressive (4%) disorders based on DSM-IV criteria.

Regarding the patient characteristics mentioned in Table 1, there were no statistically significant differences between patients who were treated by means of mastectomy as compared to breast-conserving therapy (results not shown).

### Needs and preferences

Information on long-term effects and side effects of treatment and prognosis were considered as very important (Table 2

**Table 2:** Needs and preferences during the follow-up visits (%)

	**Not important/do not want this** (%)	**Not very important/do not care** (%)	**Very important/I certainly want this** (%)
**General topics**			
*How much would you like the following topics to be part of the follow-up visit?*			
Information on the long-term effects of treatment	2.5	13.50	84.00
Information on own prognosis	6.3	8.9	84.80
Information on life rules after a diagnosis of breast cancer (e.g. nutrition)	7.5	26.30	66.30
Information on side effects of treatment	3.7	9.9	86.40
Additional investigations (e.g. X-ray and blood tests)	6.6	11.80	81.60

**Specific topics**			
*In how far you would like to talk about the following subjects during the follow-up visits?*			
Prevention of breast cancer	13.20	14.50	72.40
Heredity factors	18.40	13.20	68.40
Changes in the untreated breast	20.20	14.90	64.90
Feeling fatigued	25.00	22.40	52.60
Pain (e.g. nerve pain)	26.00	27.40	46.60
Fear	38.20	21.10	40.70
Nutrition	37.80	35.10	27.10
Use of OACs or HRTs^a^	39.10	11.60	49.30
Breast reconstruction (if applicable)	50.00	24.20	25.80
Acceptation by family and friends	52.80	27.80	19.40

*How much you would like the following topics to be part of the follow-up visit?*			
Information on breast cancer self-help groups	40.50	40.50	19.00
Consultation with psychologist or psychiatrist	53.80	38.50	7.7
Consultation with hospital social worker	44.30	39.20	16.50
Consultation with pastoral care provider	59.00	34.60	6.4

**Organisation**	**Every year** (%)	**Every 6 months** (%)	**Every 3 months** (%)
How often would you prefer to attend routine control visits?	16.20	58.80	25.00
	**5 years (%)**	**10 years (%)**	**Lifelong (%)**

For how long would you prefer to attend routine control visits?	12.10	22.00	65.90

	**General practitioner (%)**	**Specialised nurse (%)**	**Hospital doctor (%)**
By whom should the follow-up be performed?	7.60	6.30	86.10

a OA=oral anticonceptiva; HRT=hormone replacement therapy.

). Patients preferred additional investigations (like X-ray and blood tests) to be part of routine follow-up visits. Regarding more specific topics, being able to discuss prevention of breast cancer, hereditary factors, and changes in the untreated breast were considered as very important by most patients. More than half of the patients preferred lifetime follow-up, twice a year, performed by a hospital doctor.

### Determinants of needs and preferences

Higher information needs were associated with: receiving adjuvant hormonal therapy or chemotherapy, feeling more nervous anticipation, perceiving more disadvantages of follow-up, more fear of recurrence, and with higher scores on the HADS-anxiety or HADS-depression scales (Table 3

**Table 3:** Determinants of needs and preferences (Spearman's rho)

	**General topics**	**Specific topics**	**Preference for additional investigations**	**Preferred follow-up frequency**	**Preferred follow-up time**	**Preferred follow-up provider**
Age	−0.35**	−0.32**	**−**0.16	**−**0.19	**−**0.1	0.01
Stage of breast cancer	0.05	0.18	0.16	**−**0.05	0.04	0.17
Therapy	0.07	**−**0.14	**−**0.3	**−**0.14	**−**0.16	−0.23*
Adjuvant hormonal therapy	0.24*	0.21	0.17	0.23*	0.05	0.04
Adjuvant chemotherapy	0.25*	0.23*	0.13	0.18	**−**0.07	**−**0.01
Adjuvant radiotherapy	**−**0.06	0.22	0.13	0.29**	0.06	0.23*
Duration of follow-up in years	**−**0.05	**−**0.06	0.05	**−**0.14	0.13	**−**0.06
*Attitudes towards follow-up*						
Communication (with the physician)	**−**0.1	**−**0.03	**−**0.2	0.08	**−**0.01	0.15
Reassurance	**−**0.2	0.02	**−**0.14	0.03	0.21	0.30**
Nervous anticipation	**−**0.07	0.25*	0.10	**−**0.02	0.13	**−**0.04
Specific perceived disadvantages of follow-up	0.10	0.31*	0.15	**−**0.15	**−**0.12	−0.47**

Expected benefits from follow-up	**−**0.01	0.01	**−**0.02	0.18	0.04	0.04

*Ware's Patient Satisfaction Questionnaire III*						
Technical competence	**−**0.03	**−**0.05	**−**0.15	**−**0.16	**−**0.06	0.03
Interpersonal aspects	**−**0.18	−0.26*	**−**0.17	**−**0.08	0.02	0.12
Access to care	**−**0.04	**−**0.18	**−**0.14	**−**0.05	**−**0.06	0.18
Total score	**−**0.12	**−**0.17	**−**0.16	**−**0.08	**−**0.03	0.15

*Quality of life and psychological functioning*						
Fear of recurrence	**−**0.05	0.26*	0.21	0.30**	0.15	0.19
Quality of life on a visual analogue scale	−0.26*	**−**0.18	**−**0.11	**−**0.08	0.07	**−**0.05
Quality of life on a verbal scale	**−**0.05	−0.28*	**−**0.14	**−**0.15	**−**0.07	**−**0.06
HADS-anxiety	0.08	0.52***	0.23*	0.14	0.14	0.04
HADS-depression	**−**0.18	0.29*	0.24*	0.17	0.04	0.06

* *P*<0.05,

** *P*<0.01,

*** *P*<0.0001.

). Lower informational needs were related to higher age, higher quality of life score, and more satisfaction with interpersonal aspects (interpersonal manner and time spent with physician).

Women with higher scores on the HADS-anxiety or HADS-depression scales had a stronger preference for additional investigations (such as X-ray and blood tests) to be part of routine follow-up visits. Women receiving adjuvant hormonal therapy and adjuvant radiotherapy and women with a higher fear of recurrence preferred a more intensive routine follow-up schedule. A greater sense of reassurance from follow-up and less perceived disadvantages related to follow-up were connected to a more frequent preference for a hospital doctor as follow-up provider. Women who had had breast-conserving therapy and women who had had adjuvant radiotherapy more often preferred a hospital doctor as follow-up provider.

In multivariate analyses, satisfaction with interpersonal aspects (interpersonal manner and time spent with physician), the score on the HADS-anxiety scale, the adjuvant hormonal or radiotherapy, and the perceived disadvantages remained as independent predictors of needs and preferences (Table 4

**Table 4:** Determinants of needs and preferences (multivariate analyses, *β*′s)

	**General topics**	**Specific topics**	**Preference for additional investigations**	**Preferred follow-up frequency**	**Preferred follow-up provider**
Age	−0.22	−0.19	n.i.	n.i.	n.i.
Therapy	n.i.	n.i.	n.i.	n.i.	−0.16
Adjuvant hormonal therapy	0.14	n.i.	n.i	0.23*	n.i.
Adjuvant chemotherapy	−0.01	.0.13	n.i.	n.i.	n.i.
Adjuvant radiotherapy	n.i.	n.i.	n.i.	0.25*	0.28

*Attitudes towards follow-up*					
Reassurance	n.i.	n.i.	n.i.	n.i.	0.04
Nervous anticipation	n.i.	0.08	n.i.	n.i.	n.i.
Specific perceived disadvantages of follow-up		0.01	n.i.	n.i.	−0.33**

*Ware's Patients Satisfaction Questionnaire III*					
Interpersonal aspects	n.i.	−0.22*	n.i.	n.i.	n.i.
*Quality of life and psychological functioning*					
Fear of recurrence	n.i.	0.07	n.i.	0.13	n.i.
Quality of life on a visual analogue scale	−0.13	n.i.	n.i.	n.i.	n.i.
Quality of life on a verbal scale	n.i.	−0.2	n.i.	n.i.	n.i.
HADS-anxiety	n.i.	0.38**	0.14	n.i.	n.i.
HADS-depression	n.i.	−0.21	0.12	n.i.	n.i.

* *P*<0.05,

** *P*<0.01.

a n.i.=variable not included in the multivariate model.

).

## DISCUSSION

We analysed which needs there were regarding information and preferences for additional investigations and organisation of follow-up care, among women who participated in a routine follow-up programme after treatment for primary breast cancer. Information on long-term effects and side effects of treatment and prognosis was considered very important, as well as discussing prevention of breast cancer, hereditary factors, and changes in the untreated breast. Patients preferred additional investigations (like X-ray and blood tests) to be part of routine follow-up visits. More than half of the patients preferred lifetime follow-up, twice a year and performed by a hospital doctor. That patients preferred additional investigations (like X-ray and blood tests) to be part of routine follow-up visits has also been reported in other studies (Muss *et al*, 1991). Related to this, more than half of the patients in our study preferred lifetime follow-up and have high expectations from follow-up. Most patients (88%) think that early detection of distant recurrences will contribute to their cure.

Patients needs and preferences are in contrast with the available evidence in the literature on the value of diagnostic tests for the detection of asymptomatic metastases. This finding stresses the point that patients need to be educated about the effectiveness of follow-up examinations. It is now generally accepted that routine surveillance for the early detection of breast cancer recurrence at distant sites does not improve survival (GIVIO Investigators, 1994; Rosselli *et al*, 1994; Joseph *et al*, 1998; Palli *et al*, 1999). The fact that patients expected to have more chance of survival by performing more tests and detect metastases at an early stage means that they lack information on the primary goals of follow-up. Also, the chance of recurrence is decreasing by the widespread use of adjuvant therapy and there is no need for lifetime follow-up. This survey reveals shortcomings in our patient education at that time. Patients were not counselled by a breast-care nurse and there was no structured written information on these topics available. A number of innovations have been introduced in our care during the last years and it would be interesting to see if a more proper education programme will have influence on the expected benefits of routine follow-up care in breast cancer patients.

Patient's needs and preferences are supposed to be related to the follow-up care that patients actually receive. The follow-up programme was based on the guidelines of the Dutch Association of Comprehensive Cancer Centers of the nineties (Vademecum Oncologie, 1992), which is merely based on expert opinion than on evidence on how often patients should be reviewed to fulfill the goals of follow-up (detection of recurrence, detection of side effects of treatment, and psychosocial support).

Contrary to the findings of other studies (Jefford and Tattersall, 2002), not all patients needed all types of information during routine follow-up, after a diagnosis of breast cancer. We found that only one patient desired all the information about her cancer and half of the patients rated less than 50% of the information as very important, suggesting that need for information decreases over time after a diagnosis of breast cancer. Indeed, Mesters *et al* (2001) also reported that the group of patients expressing no need for information increased 1 year after treatment. In our study, the median time from surgical treatment to follow-up was 3 years and ranged from 2 to 4 years, which may explain the seemingly further decrease of need for information. It would be interesting to do research on how the needs and preferences of patients change over time when the interval to their primary treatment is increasing.

Younger age was related to a greater need for information during follow-up. The relationship between age and information need among patients with a recent diagnosis of cancer was reported earlier (Cassileth *et al*, 1980; Wallberg *et al*, 2000). Less satisfaction with interpersonal aspects (interpersonal manner and time spent with physician) and a higher score on the HADS-anxiety scale were also related to higher information needs. Other studies also found that the need for information after a diagnosis of cancer was positively related to an elevated level of anxiety (Jefford and Tattersall, 2002). However, one cannot conclude from these findings that information provision will reduce anxiety levels.

For patients newly diagnosed with cancer, the priority information needs are focused on short-term effects and appear to be: details about available treatment regimes, side effects of treatment, extent of the disease, likelihood of cure and prognosis, self-care, and return to a normal lifestyle (Mills and Sullivan, 1999). Comparing these needs to those of the patients in our study, we see more information needs focused on long-term effects of a diagnosis of breast cancer.

Higher scores on the HADS-anxiety and -depression scale were related to stronger preferences for additional investigations. Receiving adjuvant hormonal or radiotherapy was related to a preferred, more intensive, follow-up schedule. This can be ascribed to the fact that, according to national guidelines, patients with a worse prognosis more often receive adjuvant therapy.

As in other studies (Morris *et al*, 1992), patients preferred follow-up to be performed by a hospital doctor. This may be explained by the fact that at the time of this study, a hospital doctor performed follow-up twice a year (due to the length of follow-up), making this the familiar situation. Results from other studies show that patients who have really experienced follow-up performed by a breast-care nurse (Earnshaw and Stephenson, 1997) or by their general practitioner are very satisfied with it (Grunfeld *et al*, 1999).

Overall, the HADS-anxiety scores (mean: 5.0 *vs* 5.1) and the HADS-depression scores (mean: 2.7 *vs* 3.4) were comparable to Dutch norm scores. In literature, high HADS scores are found among women with a recent diagnosis of breast cancer. For example, Osborne reported a prevalence of 24% of women, with scores of 11 or greater, being most likely to have had anxiety or depressive disorders based on DSM-IV criteria (Osborne *et al*, 2003). Others showed that after a diagnosis of breast cancer, psychological morbidity tended to decrease over a 12-month period (McArdle *et al*, 1996). After a year, the prevalence of anxiety and depression was not increased in long-term survivors of breast cancer who are apparently free of disease (Ellman and Thomas, 1995).

Regarding follow-up, women tend to vary in their appreciation of different aspects of follow-up (Adewuyi-Dalton *et al*, 1998; Koinberg *et al*, 2001). When introducing alternative follow-up schedules, individual patients’ information needs and preferences should be identified early and incorporated into this routine follow-up care, to target resources and maximise the likelihood that positive patient outcomes will result.
